# Patient adherence to physical activity advice (PAPA) in patients with low back pain: Study protocol for a multicentre randomized controlled trial

**DOI:** 10.1002/pri.1969

**Published:** 2022-08-17

**Authors:** Ben van Koppen, Pim Zandwijk, Henk van Mameren, Rob de Bie

**Affiliations:** ^1^ Department of Epidemiology Faculty of Health Medicine and Life Sciences Maastricht University Maastricht The Netherlands; ^2^ Caphri Research Institute for Care and Public Health Maastricht University Maastricht The Netherlands

**Keywords:** activity advice, adherence, illness perceptions, low back pain, social support

## Abstract

**Background:**

It seems that nonspecific low back pain (NSLBP) cannot be successfully treated with a single intervention of any kind. However, a positive effect of an active lifestyle including physical activities in NSLBP is reported on pain and disability. Advising regular physical activity is one of the mainstays in physical therapy treatment, appealing to patients' adherence. Many patients with low back pain do not seem to adhere to their physiotherapist's advice. In this study, the influence of social support on adherence to an activity advice will be investigated.

**Objective:**

Objective of the study is to investigate if patients with low back pain randomised to receive social support adhere better to an activity advice than patients randomised to the control group.

**Methods:**

In a randomized controlled clinical trial, in private physiotherapy practices in the Netherlands, patients ≥18 years presenting with at least a second episode of nonspecific low back pain will be randomized over two groups: activity advice and social support as an add‐on to usual care versus usual care alone. The primary outcome measure is adherence to an activity advice measured by an activity monitor at 1, 6 and 12 weeks. All other objectives will be measured by questionnaires.

**Discussion:**

This project is the first comprehensive research project to assess whether social support influences adherence to an activity advice outside the clinical setting without supervision of a healthcare provider. Where evidence of influence on social support is lacking, findings may have implications for the management of patients with low back pain. In addition, findings may enable physiotherapists to predict in which patients with low back pain an activity advice with social support makes sense.

**ClinicalTrial.gov:**

NCT02996955.

## 1 INTRODUCTION

It seems that nonspecific low back pain (NSLBP), particularly with a recurrent or persistent course, cannot be successfully treated with a single intervention of any kind (Friedly et al., 2010). A positive effect of an active lifestyle including physical activities is reported on pain and disability (Choi et al., 2010; Dahm et al., 2010; Hendrick et al., 2011; Ooijendijk, Wendel‐Vos & de Vries, 2007; Smith & Grimmer‐Somers, 2010; Staal et al., 2013; van Middelkoop et al., 2010; van Middelkoop et al., 2011; Wai et al., 2008). Therefore, advising an active lifestyle to patients seems logical, and optimal patient adherence to this advice is necessary to be successful in managing NSLBP. As clinical practitioners we experience patients' poor adherence to physical activity recommendations every day. This experience is supported by conclusions in literature that many sufferers do not seem to adhere to their physiotherapist's advice (Beinart et al., 2013; Kolt & McEvoy, 2003).

Poor treatment adherence in patients managed in physiotherapy outpatient settings is associated with low levels of physical activity, low self‐efficacy, perceived illness beliefs, depression, anxiety, helplessness, poor social support, long treatment duration, and increased pain levels during exercise (Jack et al., 2010; Medina‐Mirapeix, Escolar‐Reina, Gascón‐Cánovas, Montilla‐Herrador, & Collins, 2009; Medina‐Mirapeix, Escolar‐Reina, Gascón‐Cánovas, Montilla‐Herrador, Jimeno‐Serrano, & Collins, 2009). Besides, there is limited or conflicting evidence on the influence of clinical signs to adherence such as pain and disability, comorbidity, obesity, and number of episodes of health threat (Jack et al. 2010). To increase patient relevant outcomes as pain and disability, it is necessary to invent new, simple applicable treatment options to overcome the adherence issue. These options should influence reasons for suboptimal adherence.

Social support is known as one of the most important factors predicting physical health (Oraison & Kennedy, 2019). While various definitions of social support have been proposed in the literature, most research recognizes social support as the help perceived (i.e., emotional, practical) from others in social networks (Oraison & Kennedy, 2019). In this study, the influence of social support on adherence to an activity advice will be investigated. For the purpose of this study, it is hypothesized that organizing social support by a partner or friend will be a potential treatment option to increase adherence to a physical activity advice. This intervention fits the context of the new health definition where health is defined ‘as the ability to adapt and to self‐manage, in the face of social, physical, and emotional challenges’ (Huber, 2014).

There is evidence indicating that illness beliefs predict health behaviour and activity limitations in low back pain. To minimize negative influence of maladaptive illness beliefs on adherence to a physical activity advice in patients with low back pain (Siemonsma et al., 2013), in this randomized controlled trial, treatment of illness beliefs is added.

Because evidence on clinical barriers is conflicting and limited, influence of confounding factors, such as age, gender, education level, pain and disability, comorbidity, and patient's intention and attitude to physical activity will also be studied. It is hypothesised these factors influence adherence.

The primary objective of this study is to investigate if patients with low back pain randomised to receive social support adhere better to an activity advice than patients randomised to the control group.

Secondary objectives are; whether patients with low back pain perceiving social support during physiotherapy treatment adhere better to an activity advice compared to patients with low back pain without perceiving social support during treatment, and, if age, gender, education level, pain and disability, treatment of illness beliefs, comorbidity and attitude and intention to physical activity influences a patient's adherence to an activity advice.

## 2 METHODS

This study is a double blinded multicentre randomized controlled trial, with a concealed randomisation. This protocol was conducted according to Standard Protocol Items: Recommendations for Interventional Trials (SPIRIT) guidelines.

### 2.1 Participants

The trial will be performed in 20 private practices for physiotherapy in the Netherlands. Dutch people have access to their physiotherapist without a referral of their GP. People with an additional insurance are compensated for the costs. For this study physical inactive patients with low back pain are needed. It is hypothesized that patients having more than one episode of NSLBP are potentially more inactive compared to patients with a first episode, therefore patients with a first episode of low back pain will be excluded. Patients ≥18 years presenting with a self‐reported second episode (or more) of NSLBP are recruited for the study. Eligible patients will be recruited for the study together with their partner or friend. Patients will be excluded for the study if (1) they cannot bring a partner or friend, (2) they are unable to read, write or speak the Dutch Language, (3) they present red flags as mentioned in the Dutch low back pain guideline (Staal et al., 2013) or, (4) when presenting with a medical history of cancer, osteoporosis, rheumatoid arthritis, tuberculosis, trauma and fractures in the lumbar spine as well as recent musculoskeletal system infections (Henschke et al., 2009; Staal et al., 2013). Only physically inactive NSLBP sufferers will be included, therefore, patients adhering to the Dutch Standard Healthy Physical Activity (DSHPA) which prescribes being moderately active for at least 150 min per week will be excluded as well (Hildebrandt, Bernaards & Stubbe, 2007).

Eligibility criteria for participating physiotherapists are: (1) being a certified graduate physiotherapist, (2) being instructed and updated on the content of the study.

During the first meeting all physiotherapists will be informed about the Dutch guideline for low back pain (Staal et al., 2013) and state of the art knowledge, to provide that treatment during the study will be in accordance with the guideline. Before the second meeting participating physiotherapists will be divided into three groups: in every clinic, an Assessor Physiotherapist (AP), an Intervention Group Physiotherapist (IGP) and a Control Group Physiotherapist (CGP) is needed. The second meeting will be for AP's, IGP's and CGP's separately. The AP's will be trained in knowledge about the study, inclusion criteria, exclusion criteria, informed consent, standardized intake forms and assessments. They will be trained how to communicate about the study in a positive manner to the patient and partner/friend. It will be explained to the physiotherapists how to adhere to the allocation procedure and how to register all investigational procedures and the use of the activity monitor. Participating CGP's will be instructed on the background and rationale of the study, treatment of illness beliefs, the activity advice, and the use of the activity monitor. Participating IGP's will be instructed on the same elements with an additional short course on how to motivate the patient and their partner/friend in organizing social support. Some participating physiotherapists are students Orthopaedic Manipulative Physical Therapy at the Rotterdam University of Applied Sciences, Rotterdam, The Netherlands. They participate in this study as APs where they must conduct a management assignment as part of their Master of Science educational program.

### 2.2 Interventions

During treatment, according to the Dutch guideline for low back pain, patients in the intervention and control group are asked to walk and/or cycle outdoors in accordance with the DSHPA (Hildebrandt et al., 2007).

In the intervention group illness beliefs according to Leventhal's Common‐Sense Model of self‐regulation will be discussed with the patient and the partner or friend (Siemonsma et al., 2013). During two half‐hour consultations maladaptive beliefs and feelings about low back pain are mapped in a standardized dialogue, maladaptive beliefs are challenged, evidence‐based beliefs as an alternative are formulated, and the utility is tested to become physically active according the DSHPA (Siemonsma et al., 2013). Organization of social support by a partner or friend is added. In a standardized discussion the partner or friend of the patient will be advised to organize social support for executing the activity advice.

In the control group only the illness beliefs according to Leventhal's Common‐Sense Model of self‐regulation will be discussed with the patient and the partner or friend (Siemonsma et al., 2013).

Only when requested by the patient the intervention will be discontinued.

### 2.3 Outcomes

#### 2.3.1 Baseline information

At baseline age, gender and education level will be registered.

To assess whether comorbidity influence adherence to an activity advice the medical burden of chronic illness is assessed with the Cumulative Illness Rating Scale (CIRS) (Appendix 1). The CIRS ranges from 0 (no illness) to 52 points and is a valid and reliable scale to measure comorbidities in clinical research (de Groot, Beckerman, Lankhorst & Bouter, 2003; Fortin et al., 2005).

It is hypothesised that overweighted/obese patients are protentional physical inactive. BMI of the patient will be calculated and categorized according to the WHO criteria (WHO, 2000).

It is hypothesised that the number of recurrences of low back pain influence adherence to an activity advice negatively, therefore, the number of recurrences of periods of low back pain will be noted.

To assess whether perceived pain and/or disability influences adherence to the activity advice, the visual analogue scale (VAS) for pain and the Quebec Back Pain Disability Scale (QBPDS) for disability will be used at baseline, both of which have been validated for assessing pain and disability (Bijur et al., 2001; Boer et al., 2004; Chapman et al., 2011; Smeets, Köke Lin, Ferreira & Demoulin, 2011) (Appendix 1).

Patients who refuse to enter the study will be asked to fill in the questionnaire at baseline (Appendix 1). The refusal questionnaire (RQ) is a study specified questionnaire collecting 13 reasons of refusal for entering the study (Barnes et al., 2012; Barratt et al., 2013; Williams et al., 2007).

#### 2.3.2 Primary outcome

Adherence to the activity advice according to the DSHPA is the primary outcome measure and will be measured by a 3D accelerometer (Activ8, 2M engineering, Valkenswaard, the Netherlands). During the assessment periods at one, six‐ and 12‐week patients are instructed to wear the Activ8 every day during one week. The number of days per week patients have walked or cycled according to the advice will be counted. Cut‐off for adherence will be set at a minimum of 5 times per week of at least 30 min walking and/or cycling. The Activ8 system is a valid instrument to quantify body postures and motions (Fanchamps et al., 2018). For this study only the walking and cycling activities recorded by the monitor will be used for analyses.

#### 2.3.3 Secondary outcomes

The Social Support Scale is a study specific questionnaire and assesses the rate of social support perceived by the patient and performed by the partner (Appendix 1). This questionnaire must be filled in by the patient and the partner after the investigation period and has dichotomous outcomes.

The Dutch Illness Perceptions Questionnaire Short‐form (DIPQ‐S) assesses patients' and partner/friends' illness beliefs (Appendix 1). Eight items about illness beliefs are scored on a 0–10‐point scale, one item is an open question. The questionnaire has good reliability and validity (Broadbent et al., 2006; de Raaij, Schröder, Maissan, Pool & Wittink, 2012; Leysen et al., 2015). The DIPQ‐S will be filled in at baseline, 1, 6 and 12 weeks follow up by the patient and at baseline, 1 and 12 weeks follow up by the partner or friend.

The module attitude and intention (MAI) of The Knowledge, Attitude, Self‐efficacy and Self‐regulation, Exercise and Social Support for Sports and Exercise Questionnaire will be used to assess attitude and intention to be physically active (Wink, van Woerkom & Renes, 2006). Attitude (6 items) can be scored from 6 (most negative) to 42 points (most positive), while intention (3 items) can be scored from 3 (most negative) to 21 points (most positive). Validity of this questionnaire is unknown, but it was used in the FLASH! Campaign from the Dutch Institute for Sport and Exercise in 2003–2006 (Appendix 1). This questionnaire will be filled in at baseline and after 12 weeks by the patient and partner or friend.

### 2.4 Timeline

Eligible patients will be recruited for the study together with their partner or friend by the assessing physiotherapist and will receive information about the study. If patients and partners or friends decide after 1 week of consideration they want to be included in the study, written informed consent will be asked to the patient and partner or friend and base‐line assessment of the patient and partner or friend will be performed (Appendix 1). After base‐line assessment the assessor explains to the patient that further treatment will be performed by an IGP or CGP. The patient will be contacted within 2 days for an appointment with either the IGP or CGP. The patient and partner/friend will be instructed to come both to the next two appointments. The assessor contacts the randomization centre. The randomisation centre allocates the patient for IGP or CGP and informs the assessor. The assessor instructs the IGP or CGP to make the next appointments with the patient and their partner/friend.

After the first intervention on illness beliefs with or without organizing social support, treatment will be applied according to the Dutch guideline for low back pain (Staal et al., 2013). In both groups' patients are instructed in executing the activity advice in accordance with the DSHPA and to wear a move monitor for 1 week (Hildebrandt et al., 2007). In the group in which the social support is given, the partner/friend will be asked to make a week schedule of times, activities, and contact‐control moments for the coming months.

**FIGURE 1:**
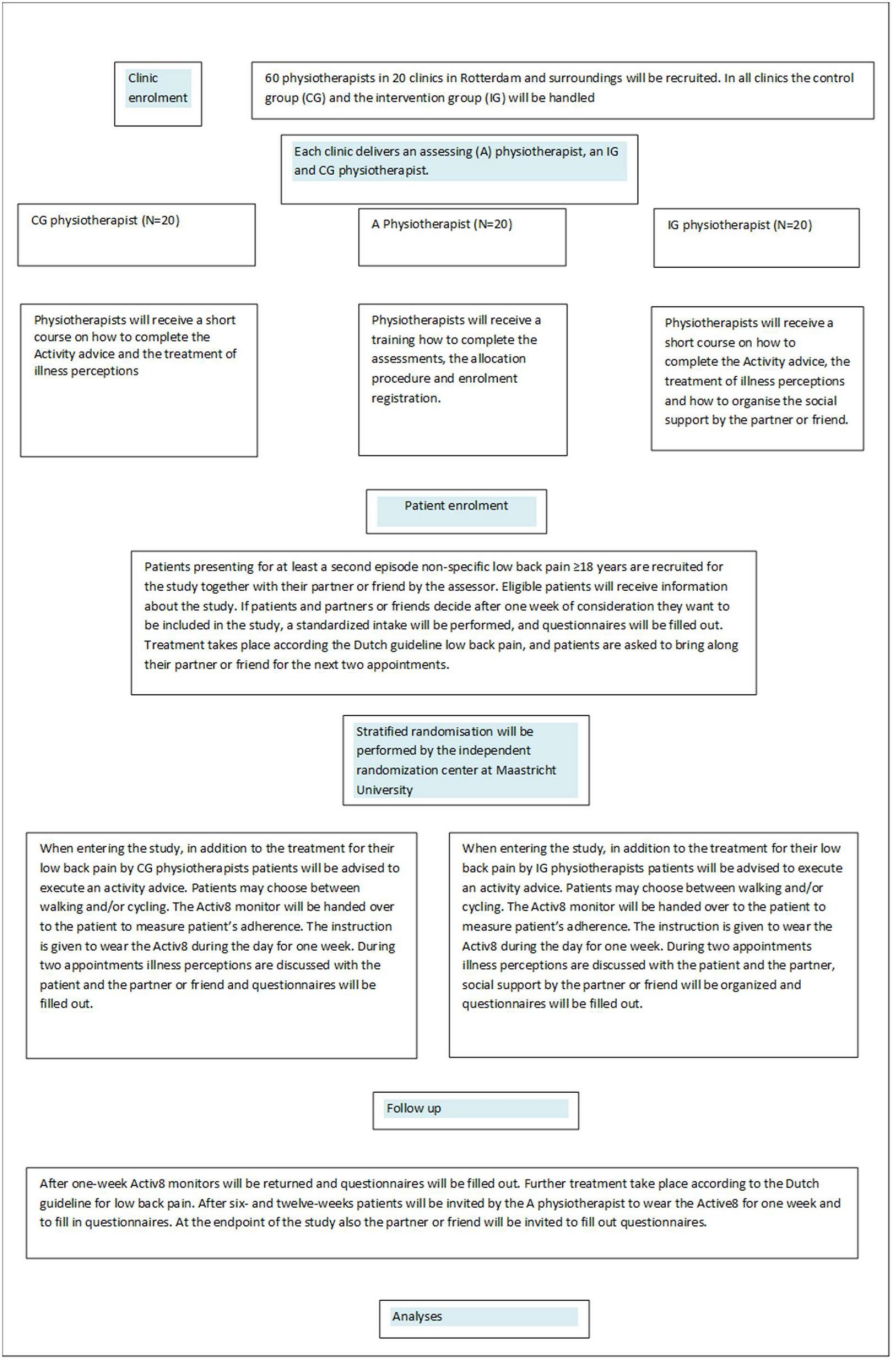
Flow diagram

At the end of the week the activity monitors will be returned, and questionnaires will be filled out. The second intervention on illness beliefs with or without organizing social support takes place and treatment will be applied according to the Dutch guideline for low back pain (Staal et al., 2013). After six‐ and 12‐weeks patients will be invited by the assessors to wear the Active8 for one week and to fill in the questionnaires. During the study period the IGP contacts the partner/friend in week 6 and 10 to evaluate whether social support was given. At the last appointment also the partner or friend will be invited and asked to fill in questionnaires. Enrolment of the study is shown in the flow diagram (Figure 1).

### 2.5 Sample size

To determine a medium effect size of the intervention on adherence to the activity advice after 12 weeks in the intervention group compared to the control group, Power G sample size calculation is performed. With an alpha of 0.05% and a power (1‐beta) of 80%, 142 patients are needed. With an expected overall drop‐out rate of 15%, a total of 163 patients is needed.

### 2.6 Recruitment

Recruitment is scheduled from October 2016 till December 2022. To achieve adequate patient enrolment, next to own consultation of patients experiencing non‐specific low back pain by participating physiotherapists, referring health care providers, such as general practitioners located in the city of the participating practices will be informed about the study, to send patients with non‐specific low back pain to those physiotherapists.

### 2.7 Assignment of interventions

It is hypothesised that patients in the intervention group adhere better to the activity advice than patients in the control group. It is also hypothesized that patients having a BMI >25 and patients experiencing >3 episodes of NSLBP are potentially more inactive and non‐receptive to the interventions on adherence. To prevent influence of these factors on study outcomes, stratification is applied on these variables. Therefore, a concealed randomization sequence is prepared by the randomisation centre of the Maastricht University. Randomization will be performed with a block size of four, with strata for BMI (<25, >24) and number of previous episodes of low back pain (2–3, >3).

Investigators, patients, partners or friends and participating physiotherapists are blinded to the allocation and outcomes. To prevent that patient and their partners/friends of both groups will meet in the waiting room appointments of patients treated in the intervention or control group in the same private practice must be planned on different days or sections of the day. Physiotherapist are blinded for participating in the intervention group or control group. To ensure blinding of the physiotherapists, where their training is separate, it will be emphasized not to communicate about the interventions to each other during the investigation period.

### 2.8 Data management

Patient data will be coded and the key to the codes will be safeguarded by the secretary of the randomization centre of the Maastricht University. These codes are unknown and not accessible for the researchers. Outcomes of questionnaires and Activ8 will be collected by the assessing physiotherapist. Losses‐to‐follow‐up and respective reasons will be recorded. The researchers collect the data from the assessors every month and enter the data in an electronic database. Access to the data obtained in this research will be restricted to the researchers involved in the collection and analysis of the data. The handling of personal data complies with the General Data Protection Regulation (GDPR) (EU, 2018). Study files will be kept for 15 years.

### 2.9 Statistical methods

To analyse the data SPSS version 25.0 (IBM SPSS, Chicago, IL, USA) will be used. For demographic data descriptive statistics will be used and normality assumptions for the variables will be checked. An intention‐to‐treat analysis will be used for the main outcome. To correspond to the primary aim of the study, outcomes of the Activ8 will be dichotomized into adherent and not adherent patients in the intervention and control group. The Chi^2^ test will be used to investigate if there is a significant difference in the proportion of adherent patients to the activity advice between the intervention and control group after one, six and 12 weeks, and if there is a significant difference in the proportion of adherent patients between patients who experienced social support or not on the social support scale after 12 weeks. A Time Series Analysis will be used to present changes over time in perceived illness beliefs of patients and partner/friend. To understand whether illness beliefs influence adherence, Pearson's r between outcomes of the IPQ‐S at baseline and after 12 weeks and (non)adherence measured by the Active8, will be calculated, and linear regression will be used for modelling the relationship between these outcomes. To assess the correlation between attitude and intention to physical activity (MAI) and adherence at 12 weeks, Pearson's will be calculated, and linear regression will be used for modelling the relationship between these outcomes. Influence of age, gender, education level, comorbidity (CIRS), pain (VAS) and disability (QBPDS) on adherence, will be estimated by multiple regression analyses. A *p* value of <0.05 will be considered statistically significant.

### 2.10 Data monitoring

Regular walking and cycling are not associated with adverse effects for NSLBP; there is no formal proof that any of these physical activities have harmful effects. The social support and treatment of illness beliefs described in this study are designed for better outcomes of physical activity levels and have no proven negative influence on pain and disability in NSLBP sufferers. Patients in both groups receive concomitant best evidence healthcare according to the Dutch guideline for low back pain (Staal et al., 2013). Therefore, composition a data monitoring committee is not needed.

### 2.11 Harms

Spontaneously reported adverse events and other unintended effects of trial interventions or trial conduct are not expected. When harms occur, it will be registered by the assessor and reported to the METC Zuyderland‐Zuyd.

### 2.12 Ethics

The study is subject to the Medical Research Involving Human Subjects Act (WMO) and has been approved by The Central Committee on Research Involving Human Subjects (CCMO), named METC Zuyderland‐Zuyd with Approval Number NL58005.096.16.

All amendments, protocol deviations and SUSAR's will be notified to the METC Zuyderland‐Zuyd. The investigator will notify the METC Zuyderland‐Zuyd of the end of the study within a period of 8 weeks. The end of the study is defined as the last patient's last visit. In case the study is ended prematurely, the investigator will notify the METC Zuyderland‐Zuyd within 15 days, including the reasons for the premature termination. Within 1 year after the end of the study, the investigator will submit a final study report with the results of the study, including any publications/abstracts of the study, to the METC Zuyderland‐Zuyd.

This research does not receive any specific grant from funding agencies in the public, commercial, or non‐for‐profit sectors. This paper presents independent research for which no conflicts of interest are to declare.

## 3 DISCUSSION

This project is the first comprehensive research project to assess whether social support influences adherence to an activity advice outside the clinical setting without supervision of a healthcare provider. Unique in this study is that intervening on illness beliefs will be provided to patient and partner to minimize the influence of maladaptive illness beliefs on adherence. Literature mentions that intervention on illness beliefs changes outcomes of the DIPQS, however, responsiveness of the questionnaire is lacking (Broadbent et al., 2015). Despite this, it is of interest if a tendence of change over time may occur and influence outcomes of adherence.

Where evidence on clinical barriers is conflicting and limited, complementary, influence of age, gender, education level, comorbidity, attitude and intention to physical activity and perceived pain and disability on adherence will be investigated. Findings have implications for the management of patients with low back pain, enabling prediction as to which low back pain patient giving an activity advice with social support makes sense.

It is important to note the variation in how concepts of (non)adherence are operationalized in existing literature resulting in variable outcomes (Essery et al., 2017; van Koppen et al., 2020). For robust outcomes in this study an accurate definition of the activity advice is applied, mentioning frequency and duration, and outcomes of adherence are dichotomous, measured with a validated move monitor.

Strengths of the study are the concealed allocation and the randomization. To reduce the presence of observer bias, measurements will not be performed by the treating physiotherapist.

Treatment of both groups in one physiotherapy practice is preferred to overcome selection bias. This implicates that despite the standardized protocol and the strict requirements to ensure blinding, it is possible that patients and treating physiotherapist exchange information about the interventions. This may influence outcomes and is therefore a limitation of the study.

## 4 IMPLICATIONS ON PHYSIOTHERAPY PRACTICE

When effectiveness of social support on adherence in NSLBP patients is demonstrated, in a patient centred approach, it can help to achieve patients' health goals.

A simple low‐cost intervention, not requiring additional education, can be added as a treatment option to increase patient relevant outcomes.

## AUTHOR CONTRIBUTIONS

Ben van Koppen, is a first author, Pim Zandwijk is a second author and Henk van Mameren, Rob de‐Bie are guarantor of the study protocol.

## CONFLICT OF INTEREST

This paper presents independent research for which no conflicts of interest are to declare.

## ETHICS STATEMENT

All amendments, protocol deviations and SUSAR's will be notified to the METC Zuyderland‐Zuyd. The investigator will notify the METC Zuyderland‐Zuyd of the end of the study within a period of 8 weeks. The end of the study is defined as the last patient's last visit. In case the study is ended prematurely, the investigator will notify the METC Zuyderland‐Zuyd within 15 days, including the reasons for the premature termination. Within 1 year after the end of the study, the investigator will submit a final study report with the results of the study, including any publications/abstracts of the study, to the METC Zuyderland‐Zuyd.

## PATIENT CONSENT STATEMENT

Not applicable.

## PERMISSION TO REPRODUCE MATERIAL FROM OTHER SOURCES

Not applicable.

## STUDY REGISTRATION

The study is registered at ClinicalTrial gov NCT02996955.

## Supporting information

Supplementary Material

## Data Availability

Data sharing is not applicable to this article as no new data were created or analyzed in this study.
