# Supplementary material for: Patient adherence to physical activity advice (PAPA) in patients with low back pain: Study protocol for a multicentre randomized controlled trial
Source: Physiother Res Int. 2022 Aug 17;27(4):e1969. doi: 10.1002/pri.1969 (PMC9786652; doi:10.1002/pri.1969)
Supplement: Supplementary file 1 — Supplementary Material [file PRI-27-e1969-s001.docx]

**Appendix 1. version 1**

|  |
| --- |

**Gestandaardiseerd intake formulier**

|  |
| --- |

Intake formulier PAPA onderzoek

Patient code:

Data baseline meting

Leeftijd:

Geslacht:

Opleiding:

BMI:

Patroon: (Jones & Rivett 2004)

1. Tekens en symptomen
2. Ontstaanswijze:
3. Ontstaansfactoren:
4. Beloop:
5. Beloop beïnvloedende factoren:
6. Aantal lage rugpijn episodes:

**Social support scale**

| **Patient** |
| --- |
| Tijdens het uitvoeren van het beweegadvies heb ik **tenminste 5 dagen per week** ondersteuning van mijn partner of vriend ervaren **de afgelopen 12 weken.**  JA  NEE |

| **Partner of vriend** |
| --- |
| Ik heb mijn partner of vriend **tenminste 5 dagen per week** ondersteund in het uitvoeren van de beweegopdracht **de afgelopen 12 weken.**  JA  NEE |

**Ziekteperceptie vragenlijst (DIPQ-s)**

Patient code:

Omcirkel alstublieft bij elke vraag het getal dat uw mening het beste weergeeft

1. Hoeveel beïnvloedt uw ziekte uw leven?

0 1 2 3 4 5 6 7 8 9 10

helemaal geen invloed zeer veel invloed

2. Hoe lang denkt u dat uw ziekte zal duren?

0 1 2 3 4 5 6 7 8 9 10

een zeer korte tijd mijn hele leven

3. Hoeveel controle vindt u dat u heeft over uw ziekte?

0 1 2 3 4 5 6 7 8 9 10

helemaal geen controle zeer veel controle

4. Hoeveel denkt u dat uw behandeling kan helpen bij uw ziekte?

0 1 2 3 4 5 6 7 8 9 10

helemaal niet zeer veel

5. Hoe sterk ervaart u klachten door uw ziekte?

0 1 2 3 4 5 6 7 8 9 10

helemaal geen klachten veel ernstige klachten

6. Hoe bezorgd bent u over uw ziekte?

0 1 2 3 4 5 6 7 8 9 10

helemaal niet bezorgd zeer bezorgd

7. In welke mate vindt u dat u uw ziekte begrijpt?

0 1 2 3 4 5 6 7 8 9 10

helemaal geen begrip zeer veel begrip

8. Hoeveel invloed heeft de ziekte op uw stemming? (Bijvoorbeeld: maakt de ziekte u boos, bang, van streek of somber?)

0 1 2 3 4 5 6 7 8 9 10

Helemaal geen invloed zeer veel invloed

9. Noem s.v.p. de drie belangrijkste factoren die naar uw opvatting uw ziekte hebben veroorzaakt, in volgorde van belangrijkheid. De belangrijkste oorzaken voor mij zijn:

1.

……………................................

2.

……………................................

3.

………………………………………….

**Cumulative Illness Rating Scale (CIRS)**

0 = geen aandoening

1 = milde aandoening: geen invloed op normale activiteit, zeer goede prognose

2 = matige aandoening: invloed op dagelijks leven, behandeling nodig, goede prognose

3 = ernstige aandoening: beperkingen aanwezig, snelle behandeling noodzakelijk, prognose is wisselend

4 = zeer ernstige aandoening: levensbedreigende situatie, spoedeisende behandeling, prognose zeer ernstig

**Cardiovasculair-respiratoir systeem**

1. cardiale aandoeningen (alleen hartaandoeningen) CIRS1-------

2. vasculaire aandoeningen CIRS2-------

(bloed, vaten, beenmerg, mit, lymfe)

3. respiratoire aandoeningen CIRS3-------

(longen, bronchi, trachea onder larynx)

**KNO/oogaandoeningen**

4. kno/oogaandoeningen CIRS4-------

(ogen, oren, neus, keel, larynx)

**Gastrointestinaal systeem**

5. aandoeningen bovenste deel CIRS5-------

(slikdarm, maag, duodenum, galwegen, pancreas)

6. aandoeningen onderste deel CIRS6-------

(dunne darm, dikke darm, hernia's)

7. lever- en galweg aandoeningen CIRS7-------

**Urogenitaal systeem**

8. aandoeningen van de nieren CIRS8-------

9. Overige CIRS9-------

(ureteren, blaas, urethra, prostaat, genitalien)

**Bewegingsapparaat en huid**

10. aandoeningen van skelet, spier of huid CIRS10------

(contracturen, artrose, decubitus)

**Neuropsychiatrisch systeem**

11. neurologische aandoeningen CIRS11------

12. psychiatrische aandoeningen CIRS12------

(depressie, verslavingen)

**Generaal systeem**

13. endocriene en metabole aandoeningen, gegeneraliseerde infecties, CIRS13------

vergiftigingen (osteoporose, schildklieraandoeningen, Diabetes Mellitus)

**Totaal score (range 0-52)**

**Module Houding en Intentie**

***Houding***

Onderstaande vragen gaan over uw ideeën en gevoel ten aanzien van sporten en bewegen (tenminste matig intensief):

Ik vind tenminste 5 dagen per week 30 minuten sporten en bewegen..

onverstandig 1 2 3 4 5 6 7 verstandig

onprettig 1 2 3 4 5 6 7 prettig

zinloos 1 2 3 4 5 6 7 nuttig

Welk gevoel roept het bij u op wanneer u zich voorstelt dat u de komende 6 weken tenminste 5 dagen in de week 30 minuten zou sporten en bewegen..

onplezierig 1 2 3 4 5 6 7 plezierig

naar 1 2 3 4 5 6 7 fijn

slecht 1 2 3 4 5 6 7 goed

***Intentie***

De volgende drie vragen gaan over of u van plan bent te sporten en bewegen (tenminste matig intensief)

Ik **ben van plan** de komende 6 weken tenminste 5 dagen per week minimaal 30 minuten te sporten en bewegen.

helemaal mee oneens 1 2 3 4 5 6 7 helemaal mee eens

Ik **ga proberen** om de komende 6 weken tenminste 5 dagen per week minimaal 30 minuten te sporten en bewegen.

helemaal mee oneens 1 2 3 4 5 6 7 helemaal mee eens

Ik **verwacht** de komende 6 weken tenminste 5 dagen per week minimaal 30 minuten te sporten en bewegen.

helemaal mee oneens 1 2 3 4 5 6 7 helemaal mee eens

**VAS pijn**

Geef op deze lijn aan hoeveel pijn u voelt

0 = geen pijn 10 = maximale pijn

**Quebec Back Pain Disability Scale**

Naam: _________________________________

datum: _________________________

Geb. datum:_________________________

|  | Totaal  geen  moeite | Nauwe-lijks  moeite | Enige  moeite | Veel  moeite | Zeer  veel  moeite | Niet  in  staat |
| --- | --- | --- | --- | --- | --- | --- |
| 1. Opstaan uit bed | **0** | **1** | **2** | **3** | **4** | **5** |
| 2. De hele nacht slapen | **0** | **1** | **2** | **3** | **4** | **5** |
| 3. Omdraaien in bed | **0** | **1** | **2** | **3** | **4** | **5** |
| 4. Auto rijden | **0** | **1** | **2** | **3** | **4** | **5** |
| 5. 20-30 minuten (achter elkaar) staan | **0** | **1** | **2** | **3** | **4** | **5** |
| 6. Enkele uren in een stoel zitten | **0** | **1** | **2** | **3** | **4** | **5** |
| 7. Een trap oplopen | **0** | **1** | **2** | **3** | **4** | **5** |
| 8. Een klein eindje lopen (300-400 meter) | **0** | **1** | **2** | **3** | **4** | **5** |
| 9. Enkele kilometers lopen | **0** | **1** | **2** | **3** | **4** | **5** |
| 10. Naar een hoge plank reiken | **0** | **1** | **2** | **3** | **4** | **5** |
| 11. Een bal werpen | **0** | **1** | **2** | **3** | **4** | **5** |
| 12. Een eindje hardlopen (+ 100 meter) | **0** | **1** | **2** | **3** | **4** | **5** |
| 13. Iets uit de koelkast pakken | **0** | **1** | **2** | **3** | **4** | **5** |
| 14. Het bed opmaken | **0** | **1** | **2** | **3** | **4** | **5** |
| 15. Sokken of panty aantrekken | **0** | **1** | **2** | **3** | **4** | **5** |
| 16. Vooroverbuigen om bv. een badkuip of WC schoon te maken | **0** | **1** | **2** | **3** | **4** | **5** |
| 17. Een stoel verplaatsen | **0** | **1** | **2** | **3** | **4** | **5** |
| 18. Een zware deur opentrekken of openduwen | **0** | **1** | **2** | **3** | **4** | **5** |
| 19. Dragen van twee tassen met boodschappen | **0** | **1** | **2** | **3** | **4** | **5** |
| 20. Een zware koffer optillen en dragen | **0** | **1** | **2** | **3** | **4** | **5** |

Onderstaande vragenlijst gaat over de manier waarop uw rugklachten uw dagelijks leven beïnvloeden. Mensen met rugklachten kunnen moeite hebben met het uitvoeren van sommige dagelijkse activiteiten. Wij willen graag weten of u moeite heeft met het uitvoeren van onderstaande activiteiten **vanwege uw rugklachten**. Voor elke activiteit is er een schaal van 0 tot 5. Wilt u bij ieder activiteit één antwoord kiezen **(geen activiteit overslaan)**, en het daarbij behorende cijfer omcirkelen.

**Vragenlijst weigering PAPA onderzoek (Refusal Questionnaire)**

Kunt u in de lijst hieronder één of meer redenen aankruisen voor uw weigering om aan het PAPA onderzoek mee te doen?

Bij voorbaat dank voor uw tijd

- 1. Ik beweeg al volgens de NNGB
- 2. Ik heb geen tijd.
- 3. Ik ben niet geinteresseerd in dit onderzoek.
- 4. Ik wil niet dat er vragen worden gesteld over persoonlijke details of mijn activiteiten.
- 5. Ik ben te oud om aan dit onderzoek mee te doen.
- 6. Ik voel me niet goed genoeg om met dit onderzoek mee te doen.
- 7. Ik kan geen partner of vriend(in) vinden die mij kan/wil ondersteunen gedurende deze onderzoekperiode.
- 8. Ik wordt niet behandeld door mijn favoriete fysiotherapeut.
- 9. Ik denk niet dat ik kan voldoen aan de eisen van de interventiegroep
- 10. Ik denk niet dat ik kan voldoen aan de eisen van de controlegroep.
- 11. Ik heb een hekel aan lopen en/of fietsen in slecht weer.
- 12. Ik ben bang dat meer bewegen mijn rugpijn doet toenemen.
- 13. Anders, nl. …………………………………………………………………………………………………………………………………….

**toestemmingsformulier proefpersoon/partner***

Behorende bij Therapietrouw aan een beweegopdracht

- Ik heb de informatiebrief gelezen. Ook kon ik vragen stellen. Mijn vragen zijn goed genoeg beantwoord. Ik had genoeg tijd om te beslissen of ik meedoe.
- Ik weet dat meedoen vrijwillig is. Ook weet ik dat ik op ieder moment kan beslissen om toch niet mee te doen met het onderzoek. Of om ermee te stoppen. Ik hoef dan niet te zeggen waarom ik wil stoppen.
- Ik geef de onderzoekers toestemming om mijn gegevens te verzamelen en gebruiken. De onderzoekers doen dit alleen om de onderzoeksvraag van dit onderzoek te beantwoorden.
- Ik weet dat voor de controle van het onderzoek sommige mensen al mijn gegevens kunnen inzien. Die mensen staan in deze informatiebrief. Ik geef deze mensen toestemming om mijn gegevens in te zien voor deze controle.
- Wilt u in de tabel hieronder ja of nee aankruisen?

| Ik geef toestemming om mijn gegevens te bewaren om dit te gebruiken voor ander onderzoek, zoals in de informatiebrief staat. | Ja ☐ | Nee☐ |
| --- | --- | --- |

- Ik wil meedoen aan dit onderzoek.

Mijn naam is (proefpersoon/ partner*): ………………………………..

Handtekening: ……………………… Datum : __ / __ / __

-----------------------------------------------------------------------------------------------------------------

Ik verklaar dat ik deze proefpersoon/ partner* volledig heb geïnformeerd over het genoemde onderzoek.

Wordt er tijdens het onderzoek informatie bekend die die de toestemming van de proefpersoon/partner* kan beïnvloeden? Dan laat ik dit op tijd weten aan deze proefpersoon/ partner*.

Naam onderzoeker (of diens vertegenwoordiger):……………………………….

Handtekening:……………………… Datum: __ / __ / __

-----------------------------------------------------------------------------------------------------------------

*De proefpersoon en partner krijgt een volledige informatiebrief mee, samen met een getekende versie van het toestemmingsformulier.*

***** doorhalen wat niet van toepassing is
